# Supplementary material for: Unusual interlayer quantum transport behavior caused by the zeroth Landau level in YbMnBi2
Source: Nat Commun. 2017 Sep 21;8:646. doi: 10.1038/s41467-017-00673-7 (PMC5608808; doi:10.1038/s41467-017-00673-7)
Supplement: Supplementary file 1 — Supplementary Information [file 41467_2017_673_MOESM1_ESM.pdf]

Description of Supplementary Files

File Name: Supplementary Information
Description: Supplementary Figures, Supplementary Notes, Supplementary Table and Supplementary References

File Name: Peer Review File

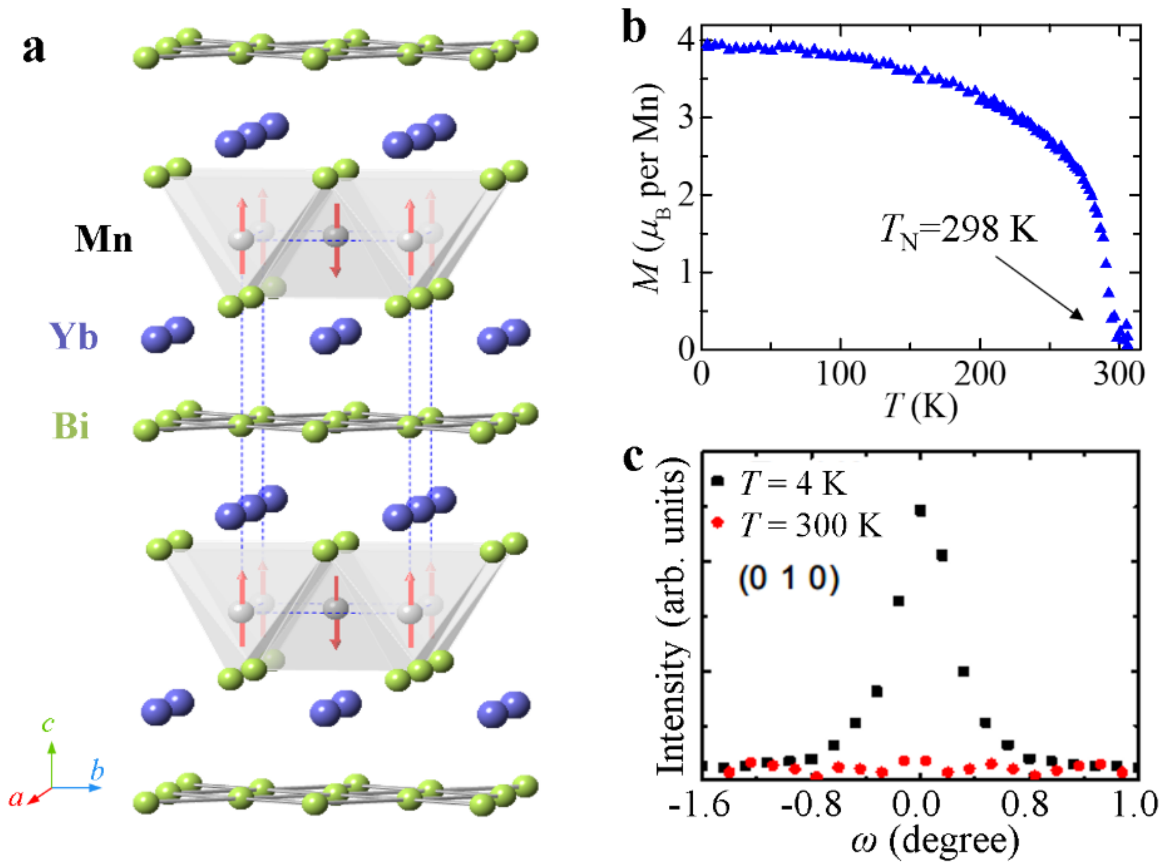

Supplementary Figure 1 | Magnetic structure characterization for $YbMnBi_2$ a, Crystal and magnetic structures determined by neutron scattering measurements. **b**, Temperature dependence of the ordered moment, measured by neutron scattering experiments. **c**, Rocking curve scan of the (0 1 0) Bragg peak. A Gaussian-shape peak is clearly observed at $T = 4$ K but not at $T = 300$ K, which is a characteristic of magnetic diffraction. Representational analysis using the BasIrreps program in FULLPROF [1] suggests that the Mn spin structure depicted in Fig. 1a is symmetry compatible and the best fit to the data as indicated by the small $\chi^2 = 0.130$ (for refining both nuclear and magnetic structure simultaneously).

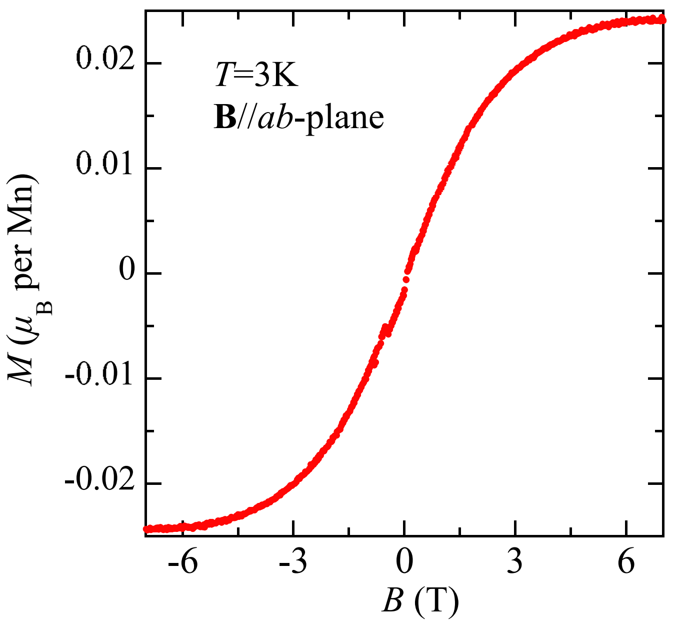

Supplementary Figure 2 | Ferromagnetic behavior seen in the isothermal magnetization measurement of $YbMnBi_2$. The saturated magnetic moment is very weak, suggesting weak ferromagnetism.

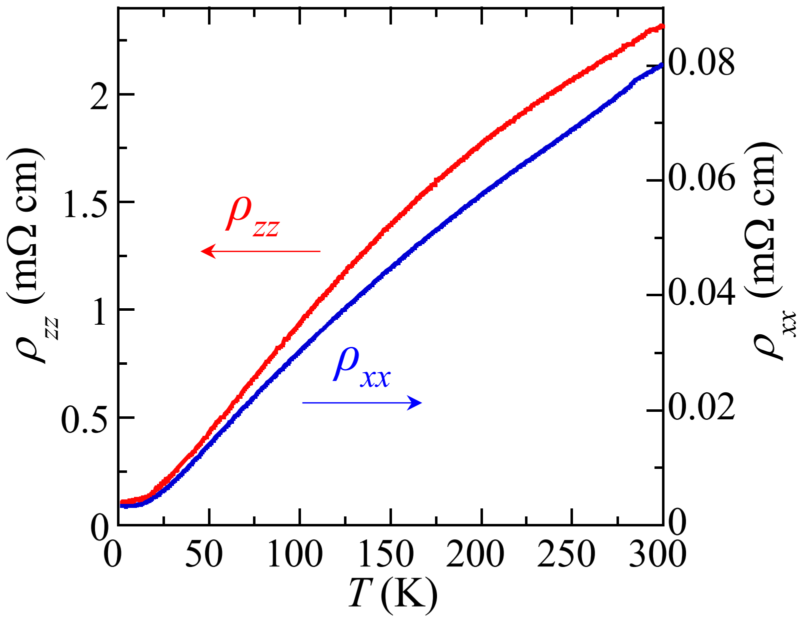

Supplementary Figure 3 | Temperature dependence of the in-plane (ρ_{xx}) and out-of-plane (ρ_{zz}) resistivity under zero field. Both ρ_{xx} and ρ_{zz} exhibit metallic behavior; the ratio of ρ_{zz}/ρ_{xx} (= 36 at T = 2 K) shows a moderate electronic anisotropy.

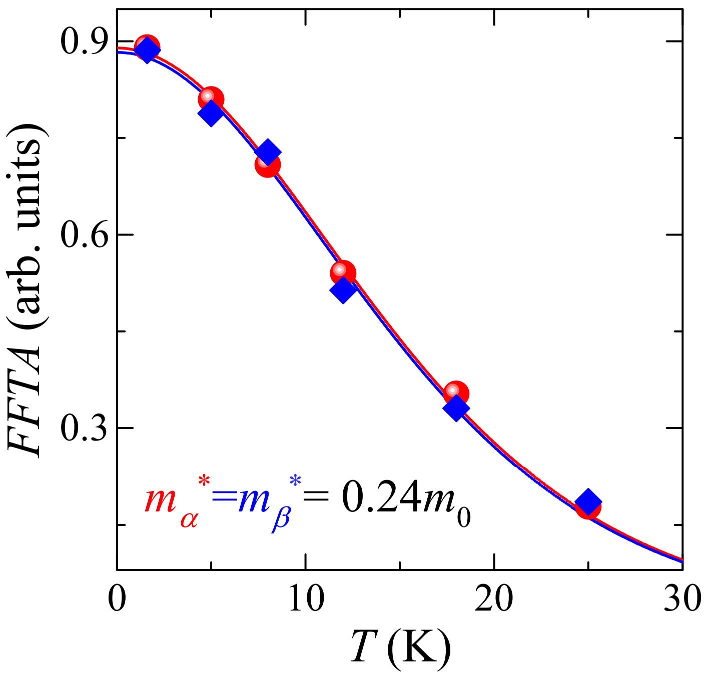

Supplementary Figure 4 | Effective cyclotron mass estimation based on the SdH oscillations of $YbMnBi_2$. The fits of the FFT amplitudes (FFTA) of the oscillatory component of ρ_{xx} by the temperature damping factor of the LK formula, which yields effective mass of $0.24m_0$ for both two frequencies.

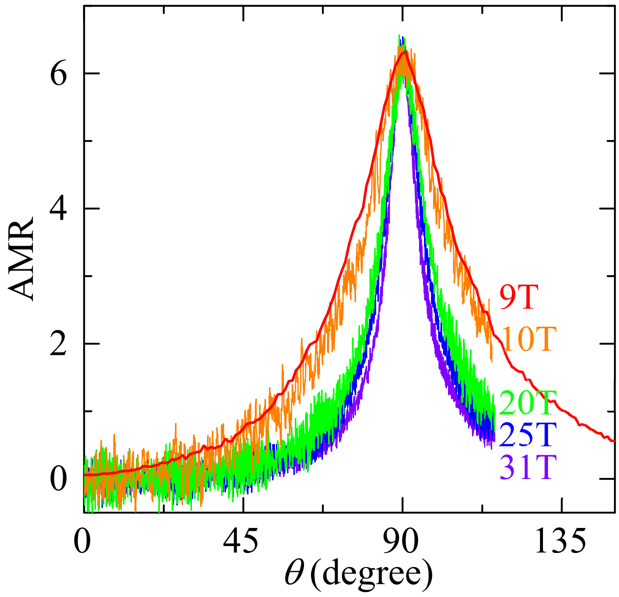

Supplementary Figure 5 | Peak width of the AMR. To better illustrate the evolution of peak width with field, the AMR data taken at 9T, 10T, 20T, and 25T are normalized to the peak of the B = 31T data, *i.e.* the low-field data are multiplied by a factor such that its peak matches with that of the 31T data. The peak width is clearly dependent on the magnetic field, inconsistent with the scenario of the coherent peak [2,3].

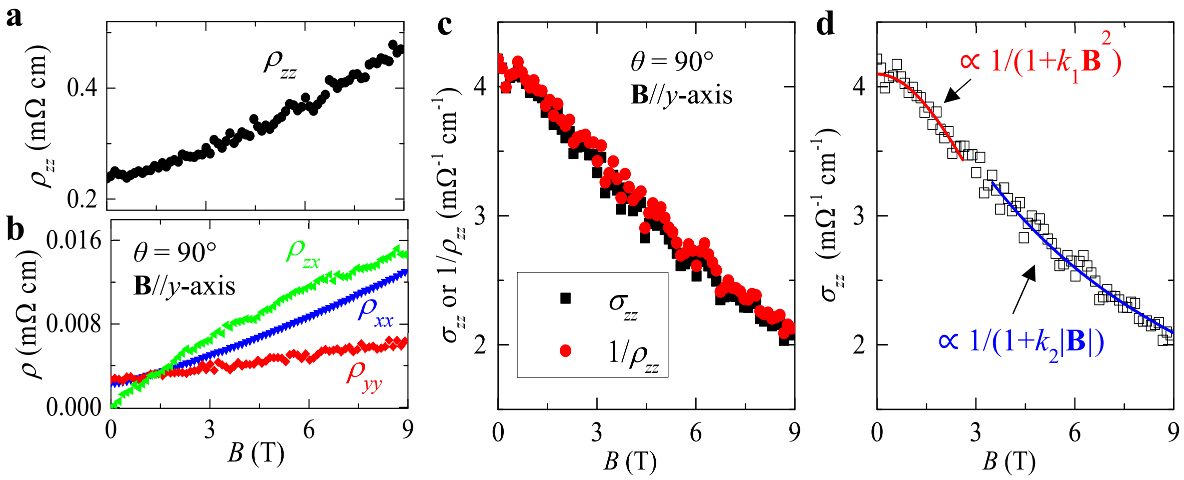

Supplementary Figure 6 | Magnetotransport measurement for $\theta = 90°$ (B//y-axis). a, Field dependence of ρ_{zz}(**B**). **b**, Field dependence of ρ_{xx}(**B**), ρ_{yy}(**B**), and ρ_{zx}(**B**). **c**, Field dependence of ρ_{zz}(**B**) and $1/\sigma_{zz}$(**B**). σ_{zz}(**B**) is obtained through tensor conversion using equation (10) (see Methods). **d**, Fitting σ_{zz}(**B**) to $\sigma_0/(1+k_1\boldsymbol{B}^2)$ and $\sigma_0/(1+k_2|\mathbf{B}|)$ in the low (0-2.5T) and high (3.5-9T) field regions respectively.

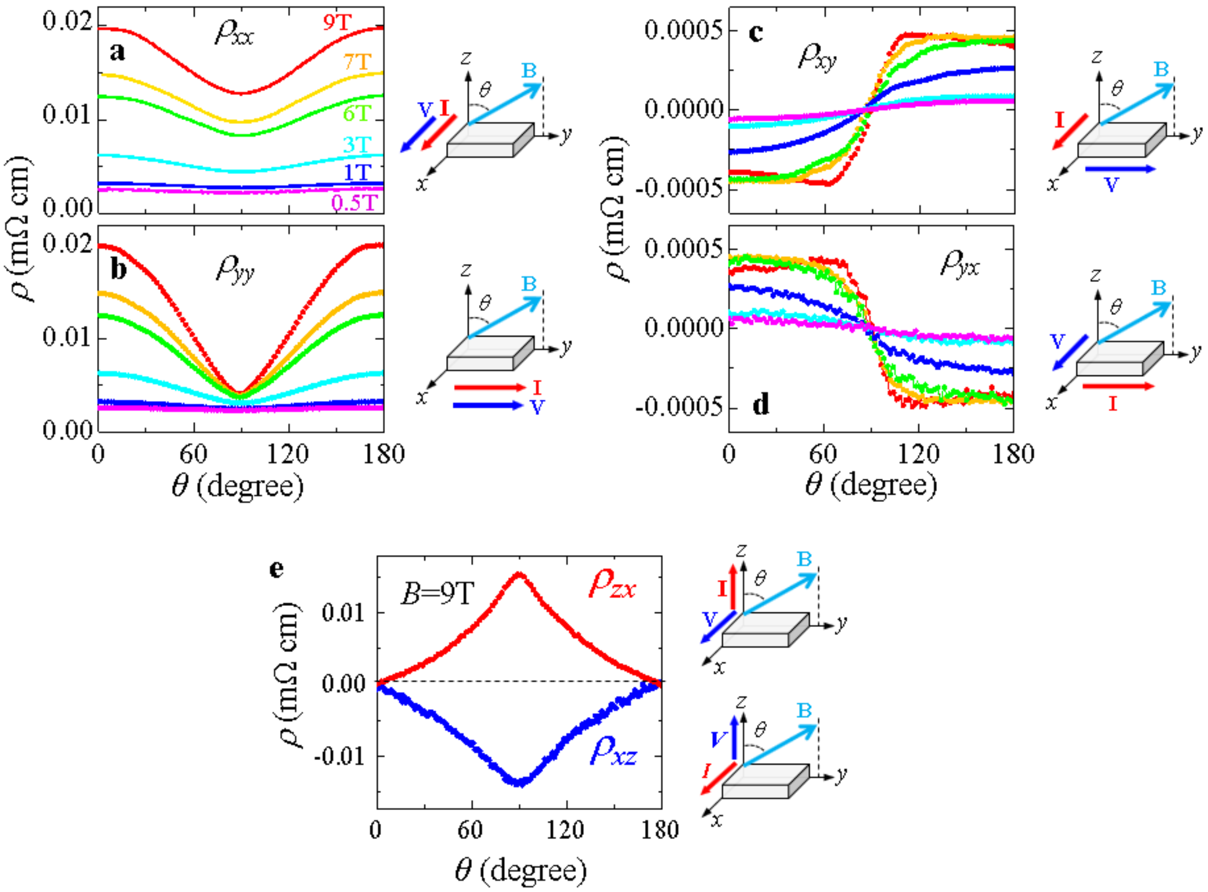

Supplementary Figure 7 | Measurements of various resistivity tensor elements for $YbMnBi_2$. a-d, Angular dependence of resistivity tensor elements (a) ρ_{xx}, (b) ρ_{yy}, (c) ρ_{xy}, (d) ρ_{yx} at different fields from 0.5 to 9T and 2K. The experimental setup for each type of measurement is shown in the right of each panel. **e,** Angular dependence of ρ_{xz} and ρ_{zx} at 9T and 2K.

Supplementary Note 1 | Exclusion of other possible mechanisms for the unusual AMR anisotropy observed in $YbMnBi_2$

Although there are several known mechanisms which can result in unusual anisotropy in magnetoresistance, none of them can offer reasonable interpretation for our observed magnetoresistance anisotropy shown in Fig. 2c. We first considered the spin scattering mechanism since $YbMnBi_2$ shows an antiferromagnetic order near room temperature. However, the spin scattering in antiferromagnetic states generally results in a sine square dependence in AMR [4], clearly inconsistent with our observation in the high field range. Second, we have examined the magnetoresistance anisotropy caused by the quantum interference effects, *i.e.* weak localization (WL) and weak antilocalization (WAL). For WAL, a sharp dip in AMR near $\theta=0^{\circ}$ is expected in our experiment setup due to quick suppression of quantum interference by the transverse field [5], which is not seen in our AMR data shown in Fig. 2b. In the case of WL, though a sharp resistance peak around $\theta = 90^{\circ}$ is expected [6], our observed positive magnetoresistance (Fig. 2b) is contradictory to the negative magnetoresistance expected for WL. Another possible mechanism we have considered is the "coherent peak" originating from the formation of small closed [2] or self-crossing orbits [3] on the side of the corrugated Fermi surface under in-plane field (B_{xy}), as seen in the Dirac semimetal $SrMnBi_2$ [7]. However, this geometric effect should lead to a resistivity peak with a field-independent width, inconsistent with our observation of the broadening of the peak with increasing the field (see Supplementary Fig. 5). Moreover, the evolution of AMR(θ) from a sharp peak near $\theta = 90^{\circ}$ to a sine-square dependence with decreasing magnetic field does not fit to any of the mechanisms discussed above.

Supplementary Note 2 | Resistivity-conductivity conversion and Hall resistivity anomalies

For the in-plane magnetic field ($\theta = 90°$, **B**//*y*-axis), the tensor elements $\rho_{xy} = \rho_{yx} = 0$, while ρ_{zz}, ρ_{xx}, ρ_{yy}, and ρ_{zx} can be experimentally measured, as presented in Supplementary Figs. 7a-7b. Because that ρ_{zz} is orders of magnitude greater than all other tensor elements ρ_{xx}, ρ_{yy}, and ρ_{zx}, σ_{zz} is approximated to be $1/\rho_{zz}$ according to equation (10). This is further demonstrated in Supplementary Fig. 6c, where the $\sigma_{zz}(\mathbf{B},\theta = 90°)$ value calculated using equation (10) almost overlaps with $1/\rho_{zz}(\mathbf{B},\theta = 90°)$.

Given that the 2D Landau level quantization in $YbMnBi_2$ should be suppressed with the in-plane field (*i.e.*, $\theta = 90°$), the interlayer transport should be mostly dominated by the momentum relaxation channel of the Dirac bands. In this case, $\sigma_{zz}(\mathbf{B})$ follows the $\sigma_0/(1+k_1B_{xy}^2)$ dependence in the low field range (0-2.5T) and the $\sigma_0/(1+k_2B_{xy})$ dependence in the high field range (3.5-9T), as shown by the fits in supplementary Fig. 6d.

Additionally, $\rho_{xy}(\theta)$ and $\rho_{yx}(\theta)$ also exhibit anomalies attributable to the zeroth LL transport. As seen in supplementary Fig. 7c and 7d, $\rho_{xy}(\theta)$ and $\rho_{yx}(\theta)$ exhibit a $\cos\theta$-like dependence in the low field range (<6T), but displays sharp kinks near 70° and 110° when the field is increased above 6T. It is worth noting that the sharp increases of $\rho_{zx}(\theta)$ (Fig. 3c) and $\rho_{zz}(\theta)$ (Fig. 2c) appear in the angle range of 70°-110°, indicating the anomalous behaviors of $\rho_{zx}(\theta)$, $\rho_{zz}(\theta)$ and $\rho_{xy}(\theta)$ all have the same origin, *i.e.* the zeroth LL's contribution to transport.

Supplementary Table 1 | Lattice parameters of $YbMnBi_2$ at $T = 4$ K, obtained from single crystal neutron scattering measurements with the goodness of fit $\chi^2 = 0.130$.

Space Group *P*4/*nmm*, $a = b = 4.460$ Å, $c = 10.824$ Å						
		x	*y*	*z*	Occupancy	B_{iso}
Atom coordinates	Yb	0.000	0.500	0.73173(56)	1	0.16351(135)
	Mn	0.000	0.000	0.000	1	0.23617(338)
	Bi1	0.000	0.000	0.000	1	0.08873(150)
	Bi2	0.000	0.500	0.16592(82)	1	0.17722(151)

Supplementary References

1. Rodríguez-Carvajal, J. Recent advances in magnetic structure determination by neutron powder diffraction. *Physica B* **192**, 55-69, (1993).
2. Hanasaki, N., Kagoshima, S., Hasegawa, T., Osada, T. & Miura, N. Contribution of small closed orbits to magnetoresistance in quasi-two-dimensional conductors. *Phys. Rev. B* **57**, 1336-1339, (1998).
3. Peschansky, V. G. & Kartsovnik, M. V. Comment on ``Contribution of small closed orbits to magnetoresistance in quasi-two-dimensional conductors''. *Phys. Rev. B* **60**, 11207-11209, (1999).
4. Fobes, D., Peng, J., Qu, Z., Liu, T. J. & Mao, Z. Q. Magnetic phase transitions and bulk spin-valve effect tuned by in-plane field orientation in $Ca_3Ru_2O_7$. *Phys. Rev. B* **84**, 014406, (2011).
5. Hu, J. *et al.* Enhanced electron coherence in atomically thin Nb_3SiTe_6. *Nature Phys.* **11**, 471-476, (2015).
6. Kennett, M. P. & McKenzie, R. H. Quantum interference and weak localization effects in the interlayer magnetoresistance of layered metals. *Phys. Rev. B* **78**, 024506, (2008).
7. Jo, Y. J. *et al.* Valley-Polarized Interlayer Conduction of Anisotropic Dirac Fermions in $SrMnBi_2$. *Phys. Rev. Lett.* **113**, 156602, (2014).
